# Elevation of Inflammatory Cytokines and Proteins after Intra-Articular Ankle Fracture: A Cross-Sectional Study of 47 Ankle Fracture Patients

**DOI:** 10.1155/2021/8897440

**Published:** 2021-01-08

**Authors:** That Minh Pham, Lars Henrik Frich, Kate Lykke Lambertsen, Søren Overgaard, Hagen Schmal

**Affiliations:** ^1^Department of Orthopedics and Traumatology, Odense University Hospital, Odense, Denmark; ^2^Department of Clinical Research, University of Southern Denmark, Odense, Denmark; ^3^Department of Neurobiology Research, Institute of Molecular Medicine, University of Southern Denmark, Odense, Denmark; ^4^Department of Neurology, Odense University Hospital, Odense, Denmark; ^5^BRIDGE-Brain Research-Inter-Disciplinary Guided Excellence, Department of Clinical Research, University of Southern Denmark, Odense, Denmark; ^6^Clinic of Orthopedic Surgery, Medical Center—University of Freiburg, Faculty of Medicine, University of Freiburg, Germany; ^7^OPEN, Odense Patient data Explorative Network, Odense University Hospital/Institute of Clinical Research, University of Southern Denmark, Odense, Denmark

## Abstract

**Introduction:**

Intra-articular fractures are the leading etiology for posttraumatic osteoarthritis (PTOA) in the ankle. Elevation of proinflammatory cytokines following intra-articular fracture may lead to synovial catabolism and cartilage degradation. We aimed to compare cytokine levels in injured and healthy ankle joints, examine the longer-term cytokine levels in fractured ankles, and investigate the association between cytokine levels in fractured ankles and plasma.

**Materials and Methods:**

In this cross-sectional study, synovial fluid (SF) and plasma of forty-seven patients with acute intra-articular ankle fractures and eight patients undergoing implant removal were collected prior to surgery. We determined concentrations of sixteen inflammatory cytokines, two cartilage degradation proteins, and four metabolic proteins and compared the levels in acutely injured ankles with those of the healthy contralateral side or during metal removal. Cytokine levels in injured ankles were also compared to serum cytokine levels. Nonparametric Wilcoxon rank-sum and Spearman tests were used for statistical analysis, and a *p* value below 0.05 was considered significant.

**Results:**

Compared to the healthy ankles, the synovial fluid in ankles with acute intra-articular fracture had elevated levels of several proinflammatory cytokines and proteases (IL-1*β*, IL-2, IL-6, IL-8, IL-12p70, TNF, IFN*γ*, MMP-1, MMP-3, and MMP-9) and anti-inflammatory cytokines (IL-1RA, IL-4, IL-10, and IL-13). The levels of cartilage degradation products (ACG, CTX-2) and metabolic mediators (TGF-*β*1 and TGF-*β*2) were also significantly higher. Synovial concentrations of ACG, IL-12-p70, IFN*γ*, IL-4, and bFGF correlated with serum levels. While most of the examined synovial cytokines were unchanged after implant removal, IL-4 and IL-6 levels were upregulated.

**Conclusions:**

We show that an acute ankle fracture is followed by an inflammatory reaction and cartilage degeneration. These data contribute to the current understanding of the protein regulation behind the development of PTOA and is a further step towards supplementing the current surgical treatment. This cross-sectional study was “retrospectively registered” on the 31th October 2017 at ClinicalTrials.gov (NCT03769909). The registration was carried out after inclusion of the first patient and prior to finalization of patient recruitment and statistical analyses: https://clinicaltrials.gov/ct2/show/NCT03769909?term=NCT03769909&draw=2&rank=1.

## 1. Introduction

Up to 80% of cases of posttraumatic osteoarthritis (PTOA) in the ankle are caused by joint injury [1, 2], and the main predisposing factor is an intra-articular ankle fracture [3–5]. The current gold standard to treat unstable ankle fracture is surgery [6–8]. However, up to 36% of intra-articular fractures in the lower extremity develop osteoarthritis [9, 10]. Unlike degenerative osteoarthritis (OA), PTOA has a known time of onset, allowing early intervention. Recent studies indicate that the initial inflammatory response following intra-articular fracture may lead to synovial catabolism and cartilage degradation [11–13], but this has so far been ignored in standard therapy.

Elevations of proinflammatory cytokines such as interleukin (IL)-1*β*, tumor necrosis factor (TNF), IL-6, and IL-8 have been documented in the early course of anterior cruciate ligament (ACL) injuries [14–17]. However, only few studies have described the upregulation of proinflammatory cytokines after intra-articular ankle fracture [12, 18, 19]. In addition, it is unclear whether cytokine levels and inflammatory cells are also elevated in whole blood after ankle fracture. Adams et al. [20] suggested that the initial inflammatory response is preserved for several months after ankle joint injury and may play an important role in cartilage degradation leading to PTOA development [21].

In this study, therefore, we aimed to identify and quantify the cytokines that are elevated in acute intra-articular ankle fractures and compare these to the levels in the contralateral healthy ankle joints. Specifically, we investigated whether synovial cytokine levels differed between the fractured and healthy ankle joints and whether cytokine levels in the fractured ankle joints correlated to plasma levels. In addition, we quantified synovial cytokine levels after implant removal. Finally, we investigated whether postinflammation was reflected in an increase in synovial immune cells.

## 2. Materials and Methods

This cross-sectional study was registered at Clinical Trials (NCT03769909) and approved by the National Committee on Health Research Ethics (J. No. S-20170139). The study is reported according to STROBE guidelines [22].

**Figure 1:**
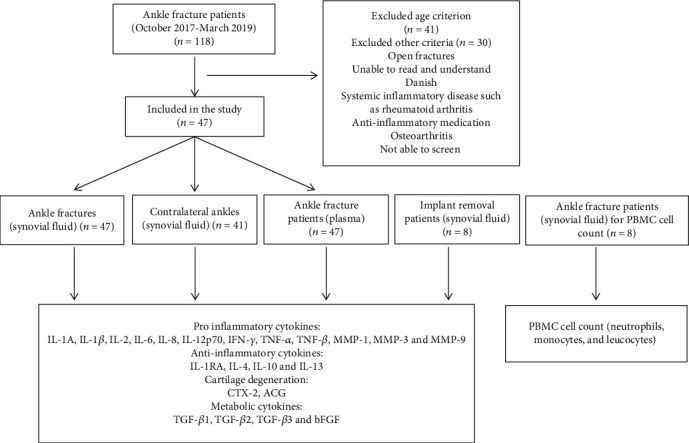
Flow diagram showing the study design.

Patients with acute intra-articular ankle fracture admitted to Odense University Hospital and Svendborg Hospital from October 2017 to March 2019 were enrolled in the study. The inclusion criteria were an acute intra-articular fracture involving the ankle joint, a need for internal or external fixation within 14 days, patient aged between 18 and 65 years and able to read and understand Danish, and written informed consent. The exclusion criteria were open fractures, associated arterial and nerve injuries, multiple injury patients with an Injury Severity Score > 15, primary or secondary infections, and systemic inflammatory diseases such as rheumatoid arthritis, anti-inflammatory medication, and injuries associated with a Charcot foot. Patients were also excluded if they had any sign of radiographic OA in the fractured or healthy contralateral ankle joint (Figure 1).

A second cohort with implant removal was recruited at our orthopedic outpatient clinic. The eligibility criteria corresponded with the primary cohort, but these patients had a consolidated ankle fracture instead of an acute fracture. Our aim with this cohort was to obtain long-term follow-up in view of the limitations of the cross-sectional study design. Patients with clinical signs of inflammation in the ankle were excluded.

For the technical reason of centralized and automatic counting, specimens used for cytokine measurement could not be used for differential cell counting. Therefore, a third cohort was included to analyze synovial cell composition in the injured ankle joints alongside leucocyte blood count.

### 2.1. Collection of Synovial Fluids and Blood Samples from Ankle Fracture Patients

Prior to surgery, synovial fluid (SF) was collected from the healthy contralateral ankle joint and then the fractured joint (*n* = 47) by puncture using the anteromedial portal. SF aspiration was performed mainly by two independent surgeons (TMP/HS). As it can be difficult to obtain sufficient volumes of SF from the ankle joint [23, 24], 5 ml saline was injected prior to aspiration in both the fractured and healthy ankle joint that included the implant removal group. SF samples were stored in 15 mL conical centrifuge tube. Blood samples for cytokine analysis and white blood cell count were collected simultaneously from the elbow vein according to the standard procedure. Within 2 hours after sampling, SF and blood samples were centrifuged at 2,000 revolutions per minute (RPM) for 15 minutes, aliquoted, and stored at -80°C until further chemiluminescence analysis.

The following epidemiological parameters were collected: age, sex, body mass index (BMI), American Society of Anesthesiologists Classification (ASA), and fracture classification of injury according to Arbeitsgemeinschaft für Osteosynthesefragen (AO) standards (43A, 43B, 43C, 44A, 44B, and 44C).

### 2.2. Chemiluminescence Analysis

SF levels of IL-1*α*, IL-1RA, IL-1*β*, IL-2, IL-4, IL-6, IL-8, IL-10, IL-12p70, IL-13, interferon gamma (IFN-y), TNF-*α*, and TNF-*β* were measured by an electrochemiluminescence immunoassay using a human customized U-Plex (Mesoscale, Rockville, MD). Matrix metalloproteinase (MMP)-1, MMP-3, and MMP-9 were measured using a human MMP-3 Plex Ultrasensitive kit (Mesoscale, Rockville, MD); transforming growth factor (TGF)-*β*1, TGF-*β*2, and TGF-*β*3 were measured using a human U-PLEX TGF-*β* Combo kit (Mesoscale, Rockville, MD); and basic fibroblast growth factor (bFGF) was measured using a Human V-PLEX bFGF kit (Mesoscale, Rockville, MD). Prior to measurement, the samples were diluted in Diluent 41, and MSD Discovery Workbench software was used for analysis (MESO QuickPlex SQ 120). Samples were run in duplex, and coefficient of variation (CV) values above 20% in individual analyses was considered high (Additional file 1). SF levels of C-terminal telopeptides of type 2 collagen (CTX-2) and Aggrecan (ACG) were analyzed by the enzyme-linked immunosorbent assay (ELISA) (MyBiosource, VersaMax™). Samples were run in duplex and performed according to the manufacturer's instructions.

### 2.3. Statistical Analysis

The *quantile-quantile* (q-q) plot tests of nearly all cytokines indicated a nonparametric pattern. To identify differences in cytokines levels between the fractured and healthy ankles, we used the paired Wilcoxon rank-sum test. The unpaired Wilcoxon rank-sum test was used to compare cytokine levels in fractured ankles and those for implant removal. The correlation analyses were performed using Spearman's test for nonparametric data and Pearson's test for parametric data. Cytokine values below the lower limit of detection (LLOD) were replaced by a value of ½ LLOD for statistical analysis. The percentage of cytokines below LLOD is presented in Additional file 1. Results are presented as medians and interquartile ranges, and a *p* value <0.05 is considered significant. All statistical analyses were performed using STATA MP 16.

## 3. Results

Data from 47 ankle fracture patients were included in the chemiluminescence analysis; these were 22 men and 25 women with mean age 42 years ± 14.4 years. The mean BMI was 27.6 ± 4.1, and nearly all fractures were classified as malleolar fractures (97.9%), with only one as a tibial plafond fracture (2.1%). SF was collected at a mean of 4.3 days postfracture, ranging from 0 to 13 days.

Eight patients undergoing implant removal were included from our outpatient clinic. In all cases, the indication for surgery was a complaint related to the implant. Removal was carried out after 9.3 months on average (range 3 to 13 months). A further nine ankle fracture patients were included for mononuclear cell (PBMC) count and had baseline characteristics as shown in Additional file 2.

### 3.1. Cytokine Levels Were Significantly Increased in Fractured Ankle Joints Compared to Healthy Contralateral Ankle Joints

Compared to levels in the healthy contralateral ankles, the ankles with acute intra-articular fracture had a statistically significant elevation of several proinflammatory cytokines, including IL-1*β*, IL-2, IL-6, IL-8, IL-12p70, TNF-*α*, IFN-*γ*, MMP-1, MMP-3, and MMP-9 (Table 1). We found a simultaneous elevation of the anti-inflammatory cytokines IL-1RA, IL-4, IL-10, and IL-13 as well as cartilage degradation products and metabolic mediators ACG, CTX-2, TGF-*β*1, and TGF-*β*2. However, no statistically significant differences were detected for IL-1*α*, TNF-*β*, bFGF, and TGF-*β*3 (Table 1).

Most of the cytokine levels in the fractured ankles did not correlate with the length of time between injury and SF aspiration (IL-1*β*, IL-2, IL-8, TNF-*α*, TNF-*β*, IL-1Ra, IL-10, IL-13, ACG, CTX-2 TGF-*β*1, and TGF-*β*2 (Table 1). However, MMP-1, MMP-3, and TGF- *β*3 had increased levels during the first two weeks postfracture. Furthermore, reduced cytokine levels were observed for IL-1*α*, IL-6, IL-12p70, MMP-9, IFN-*γ*, and bFGF (Table 1).

Overall, fracture classification was not correlated to SF cytokine levels, except for a single protease, MMP-1 (*p* = 0.002) (Additional file 3).

### 3.2. Correlation of Cytokine Levels in the Fractured Ankle Joint and Plasma

An increase in the ACG level in fractured ankles was paralleled by an increase in the ACG level in plasma (*p* = 0.03). However, the other cytokines showed no significant positive correlations (Additional file 4).

### 3.3. Comparison of Cytokine Levels in Ankle Joints of Patients Undergoing Implant Removal Vs Healthy Control Ankles and Acute Fracture Ankles

Articular levels of cytokines measured in ankles after implant removal were significantly lower than those in acute fracture ankles (Table 2). The differences were more than 100-fold for IL-1*β*, IL-8, MMP-1, MMP-3, MMP-9, IL-1RA, and IL-10. In contrast, the levels in the implant removal group were similar to those in healthy control ankles. In these two groups, the levels of several measured cytokines were below LLOD (Additional file 1). Consequently, we excluded all cytokines with a LLOD value above 50 percent and reported only results of the remaining cytokines (IL-6, IL-8, MMP-1, MMP-3, IL-1RA, IL-4, ACG, CTX-2, bFGF, and TGF-*β*1). Under this condition, we found significantly higher levels of IL-4 and IL-6 in the implant removal group compared to healthy control ankles. However, levels of MMP-1, MMP-3, and CTX-2 were significantly higher in the control ankle group (Table 2).

### 3.4. White Blood Cell Analysis of Synovial Fluid in Fractured Ankle Joints and Blood Samples

**Figure 2:**
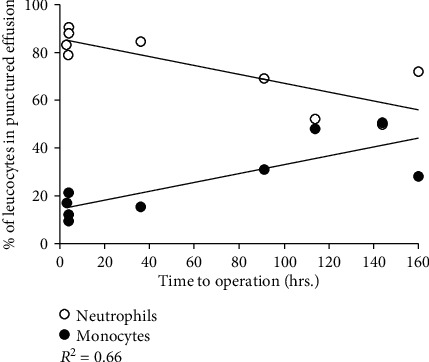
Levels of neutrophils and monocytes in synovial fluid from ankle fracture patients (*N*=8). The neutrophil level increased significantly (*P*=0.008) after injury and decreased again after 7 days (*R* value=-0.81), while the monocyte level shows the opposite changes (*R* value=0.81).

**Figure 3:**
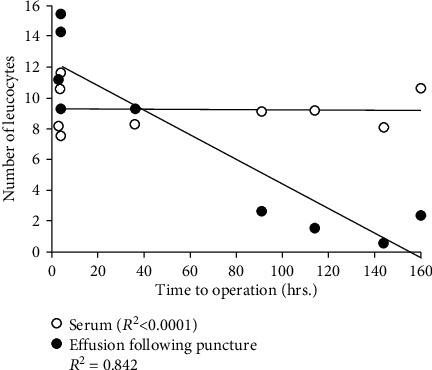
Number of leucocytes in serum and synovial fluid from ankle fracture paitents (*N*=8). The serum leucocyte level remained constant over the first 7 days after ankle fracture (*p*=0.50), while the leucocyte level in synovial fluid (effusion) decreased after ankle fracture (*p*=0.0002).

White blood cell analysis of SF in acute ankle fracture joints showed an initial upregulation of neutrophils after injury. The neutrophil level then decreased in the following days. In contrast, the monocyte level was initially low and increased over the following days (Figure 2). The number of leucocytes in serum remained constant after acute ankle joint fracture (*R*^2^ < 0.0001). However, the number of leukocytes in SF was initially high and then decreased in the following days (*R*^2^ = 0.842) (Figure 3).

## 4. Discussion

To the best of our knowledge, this is the largest study to report on cytokine levels in acute intra-articular ankle fractures using the contralateral joint for comparison and the first study to report an association between cytokine levels in the synovial fluid and plasma. We found that synovial fluid in ankles with acute intra-articular fracture had elevated levels of several pro-inflammatory cytokines (IL-1*β*, IL-2, IL-6, IL-8, IL-12p70, TNF-*α*, IFN-*γ*, MMP-1, MMP-3, and MMP-9) and of anti-inflammatory cytokines IL-1RA, IL-4, IL-10, and IL-13. In addition, synovial levels of ACG, IL-12-p70, IFN-*γ*, IL-4, and bFGF levels in acute ankle fractures correlated with the levels found in serum. Finally, we found that IL-4 and IL-6 levels were still upregulated in previously fractured joints nine months after surgery.

This study may supplement our knowledge about the regulation of the initial inflammatory cascade after acute intra-articular ankle fracture and the following longer-term conditions in the joint space. In addition, it supports the current understanding of the interaction behind the development of PTOA and offers potential avenues for supplementing the surgical treatment of fractures [11, 25, 26]. However, with a small sample size, these results need to be interpreted with caution. IL-1*β* and TNF-*α* have previously been identified as principle mediators for an acute inflammatory response after joint trauma, and they are upregulated by several cell types including chondrocytes, cells forming the synovial membrane, and infiltrating inflammatory cells such as mononuclear cells [27]. IL-1*β* and TNF-*α* upregulation stimulates cartilage matrix degradation by interfering with the synthesis of collagen type 2 and ACG and inducing a group of metalloproteinases (MMPs) that have a destructive effect on cartilage components [26]. Upregulation of IL-1*β* and TNF-*α* in synovial fluid also stimulates the synthesis of other pro-inflammatory cytokines, including IL-6 and IL-8. Production of IL-6 and IL-8 is mainly implemented by chondrocytes and macrophages and plays a major role in OA [26, 27]. Our data show that the concentrations of IL-6, IL-8, and IL-1RA increased up to 400-fold following an acute ankle fracture when compared to levels in the healthy joints. Interestingly, nearly all cytokines returned to nonmeasurable levels nine months after injury, except for IL-4 and IL-6. This is different to a previously published study by Adams et al. [20], who found that not only IL-6 levels but also IL-8 levels, MMP-1, MMP-2, and MMP-3 continued to be elevated in the ankle joint six months after injury. This may be explained by differences in patient characteristics and the methodology used, but it may also indicate that in a subgroup of patients, the imbalance of metabolism leads to long-term inflammation in the joint, resulting in cartilage degradation.

We also found elevated levels of anti-inflammatory cytokines, such as IL-1RA, IL-4, IL-10, and IL-13, in the acutely fractured ankle joints. IL-10 is a potent anti-inflammatory cytokine that has shown a chondroprotective effect in the course of OA by stimulating the synthesis of type 2 collagen and ACG [28]. Furthermore, IL-10 is involved in the inhibition of MMPs and of chondrocyte apoptosis, as well as in downregulation of IL-1*β* and TNF-*α*, by stimulating the production of IL-1RA [27]. IL-1RA can bind to the IL-1R receptor, thereby blocking the connection to IL-1*β* and indirectly inhibiting the pro-inflammatory effect of IL-1*β*.

In this study, we found that after acute ankle fracture, the levels of nearly all cytokines were elevated simultaneously after injury. While some cytokines remained at the same increased level during the first two weeks (IL-1*β*, IL-2, IL-8, TNF-*α*, TNF-*β*, IL-1RA, IL-10, IL-13, ACG, CTX-2, TBF-*β*1, and TGF-*β*2), others decreased (IL-1*α*, IL-6, IL12p-70, IFN- *γ*, MMP-9, IL-4, and bFGF), and some cytokines even increased (MMP-1 and MMP-3). This reflects the dynamic process of inflammation interfering with the cartilage metabolism. Interestingly, we found that the levels of the anti-inflammatory cytokines IL1-RA and IL-10 remained constant, while the levels of the pro-inflammatory cytokines IL-1*α* and IL-6 decreased during the first two weeks. This may indicate a downregulation of the inflammatory cascade during this period. However, some other pro-inflammatory cytokines remained elevated or even increased (MMP-1, MMP-3). This could just be because MMPs are downstream the inflammatory cascade compared to initiators such as IL-1*β*. The ratio of pro- and anti-inflammatory cytokines levels in the joint at a certain time point after injury may play an important role in the imbalance of metabolism leading to PTOA development. However, this cannot be clarified in this study.

### 4.1. Fracture Severity Does Not Correlate with Cytokine Levels in Synovial Fluid

Fracture severity has previously been correlated to increasing risk for PTOA development [29, 30]. However, this study shows nearly no correlation between fracture severity and protein levels, except for MMP-1. These findings are similar to the study of Adams et al. [20], who reported cytokine levels and fracture lines in ankle joints, while a study on tibia fractures also found also some correlation between cytokine levels and the level of fracture comminutions [25]. This could indicate that there are – not unexpectedly – also biomechanical factors that determine the outcome after fracture treatment. It is possible that the inflammatory response does not differentiate so much between severe and simple fracture patterns. Furthermore, fracture classification based on X-ray images may not be the most appropriate measurement for fracture severity and the energy level affecting the joints.

### 4.2. Correlation of Cytokines in Plasma and Ankle Joint

We believe that no previous study has examined whether cytokine elevation in acute fractured ankle joints is reflected in elevated plasma cytokine levels. It is possible that the initial impact of an ankle fracture has a systemic influence, which results in a correlation between local and global levels. This may be limited, however, as each cytokine may be secreted and degraded at other locations. In this study, we found that elevated ACG levels in the fractured ankle joint were positively correlated to plasma ACG levels. ACG has shown to be a reliable marker for cartilage degradation in previous studies [31]. The other cytokines showed no significant or compelling correlations, however, perhaps due to the timing of sample collection. We collected both blood and SF samples prior to surgery, but an elevation of cytokines in the fractured joint may not immediately cause an elevation of cytokines in the blood. Sequential blood sampling after surgery could reveal other results. The level of cytokines in the fractured ankle joint changes dynamically after joint injury. However, the ratio of pro- and anti-inflammatory cytokines levels in the joint may play an important role in the imbalance of metabolism leading to PTOA development. Evaluating the level of cytokines in the ankle joint by SF aspiration is correlated to a greater risk of joint infection and discomfort to the patients. Therefore, if a correlation of cytokines was found in the ankle joint and plasma, a blood sample may be used as a substitution. Unfortunately, in our study, only ACG was positively correlated to plasma ACG levels. A positive correlation of cytokines could indicate which cytokine might be suitable as a diagnostic biomarker in future.

### 4.3. Limitations

A limitation of this study is that we included a relatively small number of implant removal patients and ankle fractured patients for cell analysis. Furthermore, implant removal was performed only when patients had symptoms and was thus not routine. The procedure of SF aspiration from the ankle joints was quite complex and even though a limited number of surgeons performed this procedure, possible failures cannot be excluded. Finally, the cross-sectional study design meant that we were not able to monitor temporal concentrations in the ankle joints and serum. Therefore, we included the second time point related to implant removal.

## 5. Conclusions

We found elevated levels of several pro-inflammatory cytokines (IL-1*β*, IL-2, IL-6, IL-8, IL-12p70, TNF-*α*, IFN-*γ*, MMP-1, MMP-3, and MMP-9) and simultaneously elevated levels of the anti-inflammatory cytokines IL-1RA, IL-4, IL-10, and IL-13 in intra-articular ankle fractures compared to healthy contralateral joints. The level of cartilage degradation proteins ACG and CTX-2 and metabolic proteins TGF-*β*1 and TGF-*β*2 wassignificantly increased, while no differences were found for IL-1*α*, TNF-*β*, bFGF, and TGF-*β*3. Furthermore, IL-4 and IL-6 levels were still elevated up to nine months after surgery.

### 5.1. Clinical Relevance

This study contributes information about the interaction of the initial inflammatory cascade after acute intra-articular ankle fracture and the following longer-term conditions in the joint space. It supports the current understanding of the mechanism behind the development of PTOA and is a further step towards supplementing the surgical treatment of fractures.

## Figures and Tables

**Table 1:** Cytokine level in fractured ankles compared to healthy contralateral ankles.

		Fractured ankles (pg/mL) median (IQR)	Contralateral ankles (pg/mL) median (IQR)	*p* value	Cytokine ratio (fractured/contralateral)	Correlation with time after injury (0-13 days) *p* value (rho)
Proinflammatory	IL-1*α*	0.0012 (0.0132)	0.0071 (0.0000)	0.77	1.2	**0.03 (-0.318)**
	IL-1*β*	4.98 (7.96)	0.13 (0.00)	**<0.0001**	41.7	0.37 (-0.134)
	IL-2	3.30 (5.36)	0.48 (0.00)	**<0.0001**	9.1	0.53 (-0.094)
	IL-6	1914.6 (1357.2)	0.22 (0.00)	**<0.0001**	406.1	**0.01 (-0.362)**
	IL-8	490.8 (905.5)	1.06 (1.37)	**<0.0001**	245.3	0.91 (0.017)
	IL-12p70	7.07 (13.08)	0.93 (0.00)	**<0.0001**	13.5	**0.009 (-0.375)**
	TNF-*α*	7.47 (11.66)	0.4030 (0.1220)	**<0.0001**	24.5	0.52 (-0.095))
	TNF-*β*	0.000042 (0.000011)	0.000046 (0.00000)	Below LLOD	0.9	0.225 (-0.194)
	IFN-y	29.15 (44.01)	3.82 (0.00)	**<0.0001**	10.2	**0.03 (-0.322)**
	MMP-1	289571.1 (216061.4))	47.3 (1144.9)	**<0.0001**	130.2	**<0.0001 (0.683)**
	MMP-3	289571.4 (356207.6)	7966.8 (16260.4)	**<0.0001**	21.8	**0.0008 (0.505)**
	MMP-9	106324.0 (193994.9)	122.5 (4907.7)	**<0.0001**	33.2	**0.003 (-0.448)**
Anti-inflammatory	IL-1RA	1616.2 (3956.9)	0.30 (6.10)	**<0.0001**	302.9	0.25 (-0.170)
	IL-4	0.72 (1.21)	0.06 (0.00)	**<0.0001**	14.6	**0.03 (-0.319)**
	IL-10	3.08 (5.47)	0.09 (0.03)	**<0.0001**	26.7	0.26 (-0.169)
	IL-13	48.4 (49.6)	4.53 (0.00)	**<0.0001**	14.3	0.45 (-0.113)
Cartilage degradation	ACG	1829.0 (1062.0)	1084.0 (733.0)	**0.0054**	1.6	0.47 (0.124)
	CTX-2	281.1 (240.1)	161.3 (56.0)	**0.0021**	1.8	0.52 (-0.110)
Metabolic	bFGF	64.1 (149.1)	43.2 (53.4)	0.051	1.9	**0.004 (-0.472)**
	TGF-*β*1	2541.3 (4289.5)	344.6 (408.3)	**<0.0001**	6.9	0.06 (-0.317)
	TGF-*β*2	53.9 (49.5)	17.5 (26.1)	**0.0010**	1.8	0.46 (-0.126)
	TGF-*β*3	3.22 (5.96)	6.35 (0.00)	0.75	1.1	**0.0002 (0.578)**

Abbreviation: IQR: interquartile range; LLOD: lower limit of detection. Data in bold indicate a significant difference in cytokine levels. Several cytokines were significantly elevated in fracture ankles compared to contralateral healthy ankles. In addition, the cytokine level changed significantly depending on time since injury.

**Table 2:** Cytokine level in implant removal compared to fractured and healthy contralateral ankles.

		Implant removal ankles pg/mL median (IQR)	Fractured ankles pg/mL median (IQR)	Contralateral ankles pg/mL median (IQR)	Cytokine ratio (fracture/implant removal)	Cytokine ratio (implant removal/contralateral)	*p* value (fractured vs implant removal)	*p* value (implant removal vs contralateral)
Proinflammatory	IL-1*α*	0.0135 (0.000)	0.0012 (0.013)	0.0071 (0.0000)	0.6	1.9	0.070	**0.0003**
	IL-1*β*	0.67 (0.00)	4.98 (7.96)	0.13 (0.000)	185.0	2.0	**<0.0001**	**<0.0001**
	IL-2	0.71 (0.00)	3.30 (5.36)	0.48 (0.00)	6.3	1.4	**0.006**	**<0.0001**
	IL-6	0.48 (2.74)	1914.6 (1357.2)	0.22 (0.00)	72.0	5.4	**<0.0001**	**0.0019**
	IL-8	1.16 (2.73)	490.8 (905.5)	1.06 (1.37)	171.2	1.3	**<0.0001**	0.84
	IL-12p70	0.72 (0.00)	7.07 (13.08)	0.93 (0.00)	18.0	0.7	**<0.0001**	**0.001**
	TNF-*α*	0.56 (0.10)	7.47 (11.66)	0.40 (0.12)	19.4	1.2	**<0.0001**	**0.0004**
	TNF-*β*	0.000042 (0.00000)	0.000042 (0.000011)	0.000046 (0.00000)	1.0	0.9	**0.012**	**<0.0001**
	IFN-y	4.78 (0.00)	29.15 (44.01)	3.82 (0.00)	7.5	1.3	**0.008**	**<0.0001**
	MMP-1	224.7 (704.1)	289571.1 (216061.4))	47.28 (1144.9)	573.5	0.2	**<0.0001**	0.90
	MMP-3	1325.0 (2536.9)	289571.4 (356207.6)	7966.8 (16260.4)	216.5	0.1	**<0.0001**	**0.01**
	MMP-9	52.5 (2052.5)	106324.0 (193994.9)	122.5 (4907.7)	150.8	0.3	**<0.0001**	0.08
Anti-inflammatory	IL-1RA	0.47 (7.30)	1616.2 (3956.9)	0.30 (6.10)	559.9	0.5	**<0.0001**	0.56
	IL-4	0.11 (0.00)	0.72 (1.21)	0.06 (0.00)	6.0	2.2	**0.002**	**0.0001**
	IL-10	0.32 (0.00)	3.08 (5.47)	0.09 (0.03)	375.6	0.1	**<0.0001**	**0.0001**
	IL-13	4.44 (0.00)	48.35 (49.60)	4.53 (0.00)	12.8	1.0	**<0.0001**	0.06
Cartilage degradation	ACG	1033.0 (316.0)	1829.0 (1062.0)	1084.0 (733.0)	1.9	0.9	**0.020**	0.86
	CTX-2	61.8 (0.0)	281.1 (240.1)	161.3 (56.0)	5.0	0.4	**0.003**	**0.0025**
Metabolic	bFGF	24.7 (5.1)	64.1 (149.1)	43.2 (53.4)	7.4	0.4	0.091	0.23
	TGF-*β*1	53.0 (234.7)	2541.3 (4289.5)	344.6 (408.3)	34.9	0.2	**<0.0001**	**0.0085**
	TGF-*β*2	1.96 (2.83)	53.9 (49.5)	17.5 (26.1)	10.9	0.2	**<0.0001**	**0.0004**
	TGF-*β*3	0.86 (0.30)	3.22 (5.96)	6.35 (0.00)	4.7	0.2	**<0.0001**	0.75

Abbreviation: IQR: interquartile range. Data in bold indicate a significant difference in cytokine levels. Acute fractured ankles had significantly elevated levels of nearly all cytokines except from IL-1*α* and TGF-*β*1 compared to levels in implant removal ankles. The cytokine levels in implant removal and healthy contralateral ankles were overall significantly different. However, the ratio illustrated a double directional pattern. The cytokine levels in implant removal and healthy contralateral ankles were nearly all below the lower limit of detection, as illustrated in Additional file 1.

## Data Availability

All data are hosted online at OPEN (Open Patient data Explorative Network) and are available from the corresponding author on reasonable request and approval from The Regional Committees on Health Research Ethics for Southern Denmark and the National Danish Data Protection Agency https://www.sdu.dk/da/om_sdu/institutter_centre/klinisk_institut/forskning/forskningsenheder/open.Aspx.

## Abbreviations

ACG: Aggrecan
ACL: Anterior cruciate ligament
AO: Arbeitsgemeinschaft für Osteosynthesefragen
ASA: American Society of Anesthesiologists Classification
bFGF: Basic fibroblast growth factor
BMI: Body mass index
CTX-2: C-terminal telopeptides of type 2 collagen
CV: Coefficient of variation
EDTA: Ethylenediamine tetraacetic acid
ELISA: Enzyme-linked immunosorbent assay
IFN-y: Interferon gamma
IL: Interleukin
LLOD: Lower limit of detection
MMP: Matrix metalloproteinase
OA: Osteoarthritis
PTOA: Post-traumatic osteoarthritis
Q-q plot: *Quantile-quantile plot*
RPM: Revolutions per minute
SF: Synovial fluid
TGF: Transforming growth factor
TNF: Tumor necrosis factor.

## Supplementary Materials

Supplementary Materials Additional file 1: LLOD and CV values of the collected samples. Abbreviation: LLOD: lower limit of detectiossn; CV: coefficients of variation. Several cytokines in plasma, healthy contralateral ankles, and implant removal patients could not be measured accurately due to high percentage of LLOD. It may not be reasonable to compare cytokine levels between these groups. Protein levels in fractured ankles were above LLOD for all cytokines except IL-1 *α* and TNF- *β*. Data in bold indicate LLOD above 50 percent. Additional file 2: demographic characteristics. Abbreviation: M: male; F: female; BMI: body mass index; ASA: American Society of Anesthesiologists; AO: Arbeitsgemeinschaft für Osteosynthesefragen; SD: standard deviation. Additional file 3: correlation of fracture classification and cytokine levels. Data in bold indicate a significant correlation. Overall, no significant correlation was found between fracture classification and cytokine level in fractured ankles, except for MMP-1. additional file 4: correlation of cytokine levels in fractured ankles and plasma. Abbreviation: IQR: interquartile range. Data in bold indicate a significant correlation. The cytokine levels in fractured ankles were mostly not correlated with plasma levels except for ACG (positively correlated) and IL-12p70, IFN-y, IL-4, and bFGF (negatively correlated).
