# Supplementary material for: Elevation of Inflammatory Cytokines and Proteins after Intra-Articular Ankle Fracture: A Cross-Sectional Study of 47 Ankle Fracture Patients
Source: Mediators Inflamm. 2021 Jan 8;2021:8897440. doi: 10.1155/2021/8897440 (PMC7811423; doi:10.1155/2021/8897440)
Supplement: Supplementary Materials — Additional file 1: LLOD and CV values of the collected samples. Abbreviation: LLOD: lower limit of detectiossn; CV: coefficients of variation. Several cytokines in plasma, healthy contralateral ankles, and implant removal patients could not be measured accurately due to high percentage of LLOD. It may not be reasonable to compare cytokine levels between these groups. Protein levels in fractured ankles were above LLOD for all cytokines except IL-1 α and TNF- β. Data in bold indicate LLOD above 50 percent. Additional file 2: demographic characteristics. Abbreviation: M: male; F: female; BMI: body mass index; ASA: American Society of Anesthesiologists; AO: Arbeitsgemeinschaft für Osteosynthesefragen; SD: standard deviation. Additional file 3: correlation of fracture classification and cytokine levels. Data in bold indicate a significant correlation. Overall, no significant correlation was found between fracture classification and cytokine level in fractured ankles, except for MMP-1. additional file 4: correlation of cytokine levels in fractured ankles and plasma. Abbreviation: IQR: interquartile range. Data in bold indicate a significant correlation. The cytokine levels in fractured ankles were mostly not correlated with plasma levels except for ACG (positively correlated) and IL-12p70, IFN-y, IL-4, and bFGF (negatively correlated). [file 8897440.f1.docx]

| Additional file 1: LLOD and CV values of the collected samples | | | | | | | | | | |
| --- | --- | --- | --- | --- | --- | --- | --- | --- | --- | --- |
|  |  | **Fracture/contralateral ankles**  **(n­_max_= 47)** | **Fractured ankles (synovial fluid)**  **CV and LLOD** | | **Contralateral ankles (synovial fluid)**  **CV and LLOD** | | **Fractured ankles (serum)**  **CV and LLOD** | | **Implant removal (synovial fluid)**  **CV and LLOD** | |
|  |  |  | Percent of CV  above 20% | Percent below  LLOD | Percent of CV  above 20% | Percent below  LLOD | Percent of CV  above 20% | Percent below  LLOD | Percent of CV  above 20% | Percent below  LLOD |
| Pro-inflammatory | IL-1α | 47/41 | 8.5 | **83.0** | 80.5 | **90.2** | 4.4 | **77.8** | 0 | **100** |
|  | IL-1β | 47/41 | 6.4 | 8.5 | 14.6 | **90.2** | 13.3 | **91.1** | 0 | **100** |
|  | IL-2 | 47/41 | 12.7 | 23.4 | 8.5 | **97.6** | 6.7 | **97.8** | 25 | **100** |
|  | IL-6 | 47/41 | 4.3 | 0 | 2.4 | **90.2** | 6.7 | 6.7 | 12.5 | 50 |
|  | IL-8 | 47/41 | 4.3 | 0 | 0 | 12.2 | 8.9 | 37.8 | 0 | 0 |
|  | IL-12p70 | 47/41 | 14.9 | 10.6 | 4.9 | **95.1** | 4.4 | **100** | 25 | **100** |
|  | TNF-α | 47/41 | 17.0 | 6.4 | 7.3 | **97.6** | 2.2 | 22.2 | 25 | **87.5** |
|  | TNF-β | 47/41 | 25.5 | **97.9** | 34.1 | **100** | 6.7 | **95.6** | 25.0 | **100** |
|  | IFN-y | 47/41 | 14.9 | 23.4 | 26.8 | **100** | 35.5 | **55.6** | 12.5 | **100** |
|  | MMP-1 | 41/36 | 2.4 | 0 | 2.8 | 50 | 2.1 | 10.6 | 0 | 50 |
|  | MMP-3 | 41/36 | 0 | 0 | 8.3 | 0 | 2.1 | 2.1 | 0 | 0 |
|  | MMP-9 | 41/36 | 0 | 0 | 16.7 | **55.6** | 0 | 2.1 | 0 | **75** |
| Anti-inflammatory | IL-1RA | 47/41 | 10.6 | 0 | 17.1 | 34.1 | 17.8 | 2.2 | 25 | 25 |
|  | IL-4 | 47/41 | 17.0 | 14.9 | 19.5 | 4.9 | 11.1 | **91.1** | 37.5 | **87.5** |
|  | IL-10 | 47/41 | 10.6 | 4.3 | 7.3 | **92.7** | 4.4 | **64.4** | 25 | **100** |
|  | IL-13 | 47/41 | 31.9 | 4.3 | 9.8 | **100** | 6.7 | **93.3** | 12.5 | **100** |
| Cartilage  Degradation | ACG | 42/33 | 4.8 | 0 | 9.1 | 6.1 | 2.5 | 0 | 0 | 0 |
|  | CTX-2 | 38/27 | 21.1 | 18.4 | 14.8 | 18.5 | 25.6 | 24.2 | 20 | **100** |
| Metabolic | bFGF | 41/35 | 0 | 0 | 2.9 | 0 | 0 | 0 | 0 | 0 |
|  | TGF-β1 | 47/41 | 4.3 | 0 | 2.4 | 7.3 | 8.9 | 0 | 14.3 | 28.6 |
|  | TGF-β2 | 47/41 | 0 | 4.3 | 7.3 | **63.4** | 6.7 | **57.8** | 0 | **85.7** |
|  | TGF-β3 | 47/41 | 0 | **53.2** | 22.0 | **100** | 15.6 | **86.7** | 0 | **100** |
| **Abbreviation:** LLOD: Lower limit of detection; CV: Coefficients of variation.  Several cytokines in plasma, healthy contralateral ankles, and implant removal patients could not be measured accurately due to high percentage of LLOD. It may not be reasonable to compare cytokine levels between these groups. Protein levels in fractured ankles were above LLOD for all cytokines except IL-1 α and TNF- β. Data in bold indicate LLOD above 50 percent. | | | | | | | | | | |

| additional file 2: Demographic characteristics | | | | | | | |
| --- | --- | --- | --- | --- | --- | --- | --- |
|  | **Number of patients** | **Sex (M/F)** | **Age**  **(mean ± SD)** | **BMI (kg/cm^2^)**  **(mean ± SD)** | **ASA score** | **Fracture classification according to AO (43A-C, 44A-C)** | **Time from fracture to surgery**  days (percent)/ (mean ± SD) |
| Ankle fracture patients (Chemiluminescence analysis) | n=47 | 22/25 | 42.0 **±** 14.4 | 27.6 **±** 4.1 | ASA 1: 48.9%  ASA 2: 51.1% | 43C: 2.1 %  44A: 4.3 %  44B: 63.8 %  44C: 29.8 % | 4.3 ± 3.1 days |
| Ankle fracture patients (PBMC cell count ) | **n=9** | 3/6 | 38.4 ± 12.7 | 27.0 ± 4.0 | ASA 1: 55.6 %  ASA 2: 33.3 %  ASA3: 11.1 % | 43A: 0.0 %  44B: 33.3%  44C: 66.75 | 2.6 ± 2.7 days |
| Implant removal patients | n=8 | 2/6 | 49.9 ± 18.0 | 29.8 ± 4.8 | ASA1: 50.0%  ASA 2: 50.0% | 44A:12.5 %  44B: 37.5%  44C: 50.0% | 9.3 ± 3.4 (months) |
| **Abbreviation:** M: male; F: female; BMI: Body Mass Index; ASA: American Society of Anesthesiologists; AO: Arbeitsgemeinschaft für Osteosynthesefragen; SD: Standard deviation. | | | | | | | |

| additional file 3: Correlation of fracture classification and cytokine levels | | |
| --- | --- | --- |
|  |  | **P-value (rho)**  **(fracture classification vs cytokine level in fractured ankles)** |
| Pro-inflammatory | IL-1α | 0.29 (-0.158) |
|  | IL-1β | 0.29 (0.157) |
|  | IL-2 | 0.28 (0.162) |
|  | IL-6 | 0.07 (0.270) |
|  | IL-8 | 0.30 (0.156) |
|  | IL-12p70 | 0.08 (0.256) |
|  | TNF-α | 0.28 (0.161) |
|  | TNF-β | 0.89 (0.021) |
|  | IFN-y | 0.25 (0.173) |
|  | MMP-1 | **0.002 (0.464)** |
|  | MMP-3 | 0.39 (0.147) |
|  | MMP-9 | 0.70 (-0.067) |
| Anti-inflammatory | IL-1RA | 0.91 (-0.016) |
|  | IL-4 | 0.16 (0.206) |
|  | IL-10 | 0.35 (0.140) |
|  | IL-13 | 0.57 (0.084) |
| Cartilage degradation | ACG | 0.89 (-0.024) |
|  | CTX-2 | 0.96 (0.010) |
| Metabolic | bFGF | 0.20 (-0.203) |
|  | TGF-β1 | 0.24 (-0.188) |
|  | TGF-β2 | 0.55 (0.096) |
|  | TGF-β3 | 0.46 (0.120) |
| Data in bold indicate a significant correlation. Overall, no significant correlation was found between fracture classification and cytokine level in fractured ankles, except for MMP-1. | | |

| additional file 4: Correlation of cytokine levels in fractured ankles and plasma | | | | |
| --- | --- | --- | --- | --- |
|  |  | **Plasma concentration pg/mL**  **Median (IQR)** | **Fracture concentration pg/mL**  **Median (IQR)** | **P-value (rho)**  **(fractured vs serum)** |
| Pro-inflammatory | IL-1α | 0.0012 (0.0075) | 0.0012 (0.013) | 0.53 (-0.095) |
|  | IL-1β | 0.25 (0.09) | 4.98 (7.96) | 0.12 (-0.236) |
|  | IL-2 | 0.71 (0.21) | 3.30 (5.36) | 0.07 (0.269) |
|  | IL-6 | 3.42 (4.41) | 1914.6 (1357.2) | 0.06 (0.281) |
|  | IL-8 | 2.56 (3.51) | 490.8 (905.5) | 0.41 (-0.127) |
|  | IL-12p70 | 0.72 (0.28) | 7.07 (13.08) | **0.01 (-0.381)** |
|  | TNF-α | 1.90 (1.06) | 7.47 (11.66) | 0.06 (-0.288) |
|  | TNF-β | 0.000043 (0.000035) | 0.000042 (0.000011) | 0.51 (-0.100) |
|  | IFN-y | 5.70 (8.40 ) | 29.15 (44.01) | **0.006 (-0.407)** |
|  | MMP-1 | 1826.1 (3592.0) | 289571.1 (216061.4) | 0.32 (0.158) |
|  | MMP-3 | 6919.5 (4306.3) | 289571.4 (356207.6) | 0.13 (0.238) |
|  | MMP-9 | 90346.6 (59708.7) | 106324.0 (193994.9) | 0.35 (-0.149) |
| Anti-inflammatory | IL-1RA | 13.0 (23.7) | 1616.2 (3956.9) | 0.77 (0.045) |
|  | IL-4 | 0.12 (0.06) | 0.72 (1.21) | **0.01 (-0.382)** |
|  | IL-10 | 0.71 (8.83) | 3.08 (5.47) | 0.26 (-0.172) |
|  | IL-13 | 4.83 (0.95) | 48.4 (49.6) | 0.32 (0.153) |
| Cartilage degradation | ACG | 2734.5 (1411.0) | 1829.0 (1062.0) | **0.03 (0.359)** |
|  | CTX-2 | 390.1 (93.0) | 281.1 (240.1) | 0.96 (-0.008) |
| Metabolic | bFGF | 12.0 (34.0) | 64.1 (149.1) | **0.02 (-0.375)** |
|  | TGF-β1 | 2324.4 (2927.8) | 2541.3 (4289.5) | 0.93 (0.013) |
|  | TGF-β2 | 6.25 (14.67) | 53.91 (49.45) | 0.99 (0.001) |
|  | TGF-β3 | 1.16 (0.53) | 3.22 (5.96) | 0.09 (-0.255) |
| **Abbreviation:** IQR: interquartile range. Data in bold indicate a significant correlation.  The cytokine levels in fractured ankles were mostly not correlated with plasma levels except for ACG (positively correlated) and IL-12p70, IFN-y, IL-4, and bFGF (negatively correlated). | | | | |

STROBE Statement—Checklist of items that should be included in reports of ***cross-sectional studies***

|  | Item No | Recommendation |
| --- | --- | --- |
| **Title and abstract** | 1 | (*a*) Indicate the study’s design with a commonly used term in the title or the abstract (√) |
|  |  | (*b*) Provide in the abstract an informative and balanced summary of what was done and what was found (√) |
| Introduction | | |
| Background/rationale | 2 | Explain the scientific background and rationale for the investigation being reported (√) |
| Objectives | 3 | State specific objectives, including any prespecified hypotheses (√) |
| Methods | | |
| Study design | 4 | Present key elements of study design early in the paper (√) |
| Setting | 5 | Describe the setting, locations, and relevant dates, including periods of recruitment, exposure, follow-up, and data collection (√) |
| Participants | 6 | (*a*) Give the eligibility criteria, and the sources and methods of selection of participants (√) |
| Variables | 7 | Clearly define all outcomes, exposures, predictors, potential confounders, and effect modifiers. Give diagnostic criteria, if applicable (N.A.) |
| Data sources/ measurement | 8* | For each variable of interest, give sources of data and details of methods of assessment (measurement). Describe comparability of assessment methods if there is more than one group (√) |
| Bias | 9 | Describe any efforts to address potential sources of bias (√) |
| Study size | 10 | Explain how the study size was arrived at (N.A.) |
| Quantitative variables | 11 | Explain how quantitative variables were handled in the analyses. If applicable, describe which groupings were chosen and why (√) |
| Statistical methods | 12 | (*a*) Describe all statistical methods, including those used to control for confounding |
|  |  | (*b*) Describe any methods used to examine subgroups and interactions (N.A.) |
|  |  | (*c*) Explain how missing data were addressed (√) |
|  |  | (*d*) If applicable, describe analytical methods taking account of sampling strategy (N.A.) |
|  |  | (*e*) Describe any sensitivity analyses (N.A.) |
| Results | | |
| Participants | 13* | (a) Report numbers of individuals at each stage of study—eg numbers potentially eligible, examined for eligibility, confirmed eligible, included in the study, completing follow-up, and analysed (√) |
|  |  | (b) Give reasons for non-participation at each stage (√) |
|  |  | (c) Consider use of a flow diagram (√) |
| Descriptive data | 14* | (a) Give characteristics of study participants (eg demographic, clinical, social) and information on exposures and potential confounders (√) |
|  |  | (b) Indicate number of participants with missing data for each variable of interest (√) |
| Outcome data | 15* | Report numbers of outcome events or summary measures (√) |
| Main results | 16 | (*a*) Give unadjusted estimates and, if applicable, confounder-adjusted estimates and their precision (eg, 95% confidence interval). Make clear which confounders were adjusted for and why they were included (N.A.) |
|  |  | (*b*) Report category boundaries when continuous variables were categorized (N.A.) |
|  |  | (*c*) If relevant, consider translating estimates of relative risk into absolute risk for a meaningful time period (N.A.) |
| Other analyses | 17 | Report other analyses done—eg analyses of subgroups and interactions, and sensitivity analyses (√) |
| Discussion | | |
| Key results | 18 | Summarise key results with reference to study objectives (√) |
| Limitations | 19 | Discuss limitations of the study, taking into account sources of potential bias or imprecision. Discuss both direction and magnitude of any potential bias (√) |
| Interpretation | 20 | Give a cautious overall interpretation of results considering objectives, limitations, multiplicity of analyses, results from similar studies, and other relevant evidence (√) |
| Generalisability | 21 | Discuss the generalisability (external validity) of the study results (√) |
| Other information | | |
| Funding | 22 | Give the source of funding and the role of the funders for the present study and, if applicable, for the original study on which the present article is based (√) |

*Give information separately for exposed and unexposed groups.

**Note:** An Explanation and Elaboration article discusses each checklist item and gives methodological background and published examples of transparent reporting. The STROBE checklist is best used in conjunction with this article (freely available on the Web sites of PLoS Medicine at http://www.plosmedicine.org/, Annals of Internal Medicine at http://www.annals.org/, and Epidemiology at http://www.epidem.com/). Information on the STROBE Initiative is available at www.strobe-statement.org.
